# Effect of fruiting body bacteria on the growth of *Tricholoma matsutake* and its related molds

**DOI:** 10.1371/journal.pone.0190948

**Published:** 2018-02-08

**Authors:** Seung-Yoon Oh, Misong Kim, John A. Eimes, Young Woon Lim

**Affiliations:** 1 School of Biological Sciences, Seoul National University, Seoul, South Korea; 2 University College, Sungkyunkwan University, Suwon, South Korea; Woosuk University, REPUBLIC OF KOREA

## Abstract

*Tricholoma matsutake* (pine mushroom, PM) is a prized mushroom in Asia due to its unique flavor and pine aroma. The fruiting body of PM forms only in its natural habitat (pine forest), and little is known regarding the natural conditions required for successful generation of the fruiting bodies in this species. Recent studies suggest that microbial interactions may be associated with the growth of PM; however, there have been few studies of the bacterial effects on PM growth. In this study, we surveyed which bacteria can directly and indirectly promote the growth of PM by using co-cultures with PM and molds associated with the fruiting body. Among 16 bacterial species isolated from the fruiting body, some species significantly influenced the mycelial growth of PM and molds. Most bacteria negatively affected PM growth and exhibited various enzyme activities, which suggests that they use the fruiting body as nutrient source. However, growth-promoting bacteria belonging to the *Dietzia*, *Ewingella*, *Pseudomonas*, *Paenibacillus*, and *Rodococcus* were also found. In addition, many bacteria suppressed molds, which suggests an indirect positive effect on PM as a biocontrol agent. Our results provide important insights toward a better understanding of the microbial interactions in the fruiting body of PM, and indicate that growth-promoting bacteria may be an important component in successful cultivation of PM.

## Introduction

Macrofungi develop large mycelial fruiting bodies that serve as reproductive organs producing spores [1], which are an excellent natural food source for a wide range of organisms including bacteria [2, 3]. Fruiting bodies are mostly formed above-ground, although some form underground for short periods [4]. This means that bacterial communities in soil can directly or indirectly contribute to the composition of bacterial communities within fruiting bodies [5]. Pent et al. [4] showed that bacteria occurring within fruiting bodies are non-randomly selected from the surrounding soil based on their symbiotic functions or habitat requirements. Bacteria have been shown to have both positive and negative relationships with fungal fruiting bodies. For example, bacteria can trigger fruiting body formation such as that demonstrated by *Pseudomonas putida* and button mushrooms (*Agaricus bisporus*) [6]. Bacteria can also promote mycelial growth, spore germination, mycorrhiza formation [7], and provide an indirect benefit by controlling pathogens [8].

In contrast, bacteria can have negative associations with fruiting bodies. As pathogens and decomposers, bacteria often use fruiting bodies as a nutrient source [9]. For example, *Pseudomonas tolaasii* is notorious for causing brown blotch of fruiting bodies by degrading them with secreted toxins [10]. In fact, diverse bacteria that are frequently detected from fruiting bodies (*e*.*g*. *Ewingella*, *Pseudomonas*, and *Stenotrophomonas*) are known to degrade mycelia by exhibiting high chitinase activity [11–13].

The pine mushroom (PM), *Tricholoma matsutake*, is a naturally occurring edible mushroom, highly prized in Asia for its unique flavor and pine aroma [14]. The PM has not yet been successfully cultivated, so fruiting bodies are available only from forests [15]. PM fruiting body formation can be suppressed or fruiting bodies prematurely decomposed by molds inhabited in fruiting bodies [16–18]. This results in decreased quality and market prices for PM fruiting bodies. Past research on cultivating or improving yields of PM fruiting bodies has focused on identifying optimal abiotic factors such as nutrition and climatic conditions [19–22]. Recent studies, which reveal a potential close relationship between bacteria and PM [23–25], suggest that bacterial communities may also be important for successful fruiting body generation and growth [26]. Several bacteria (*e*.*g*. *Janthinobacterium*, *Pseudomonas*, and *Stenotrophomonas*) have been found in PM fruiting bodies, and the community composition appears to be different than that of the soil bacteria found in the fairy ring of PM [26, 27].

Because bacteria can influence the physiology of mushrooms [3, 8], a better understanding of how bacteria affect specifically PM growth may contribute to the successful cultivation of this mushroom in the future. In this study, we isolated bacteria from PM fruiting bodies and co-cultured them with PM isolate and with several molds associated with PM fruiting bodies to find out which bacteria directly and indirectly promote PM mycelial growth. Direct positive effects were identified by the promotion of PM mycelial growth while indirect positive effects were determined by suppression of molds isolated from PM fruiting bodies. We also characterized enzyme activity in the bacteria and molds to identify their potential roles in bacterial-fungal interactions.

## Materials and methods

### Isolation of bacteria from PM fruiting bodies

**Fig 1:**
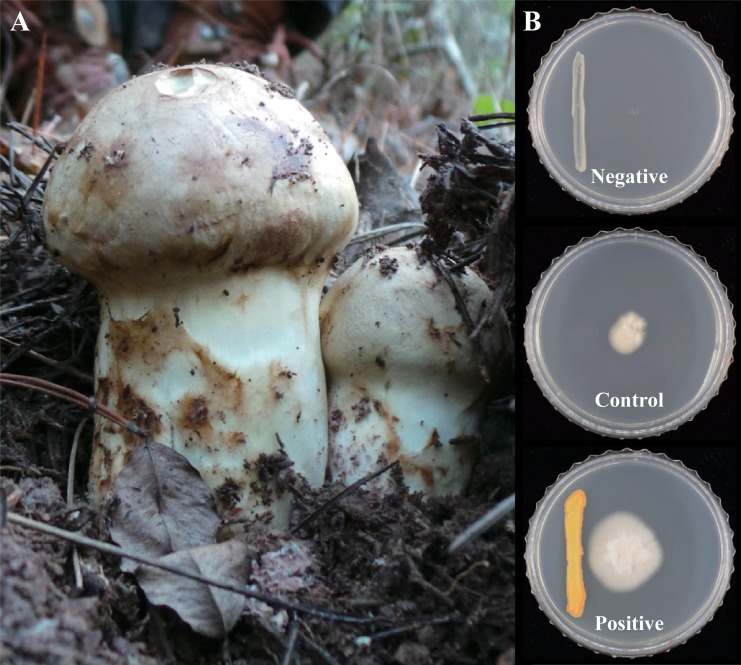
Experimental materials and design. (A) Fruiting bodies of PM at the sampling site. (B) Co-culture experiment with PM and bacteria. Control: PM not paired with any bacteria, Negative effect of bacteria: PM paired with *Mycetocola lacteus*, Positive effect of bacteria: PM paired with *Dietzia aurantiaca*.

A total of five fresh PM fruiting bodies were collected in September, 2013 from the research forest of the National Institute of Forest Science in Hongcheon (37° 41' 35"N, 127° 58' 51"E), Gangwon province, Korea (approval from the Institute). Sampling sites for each fruiting body were separated by more than 300 m. We used only fresh and immature fruiting bodies (Fig 1A) to reduce the possibility of bacterial contamination that can occur randomly due to wind, rain or other factors. Using a sterilized scalpel, we divided the fruiting bodies into 5 mm × 5 mm pieces including both the inside and outside tissues of each pileus and stipe. A total of 48 pieces from each fruiting body were placed on Tryptic soy agar (TSA; Difco, USA) and Reasoner’s 2A agar medium (R2A; Difco, USA), and incubated at 30°C for 2–7 days. Isolates were subcultured for pure culture on TSA and stored with 20% glycerol at -80°C.

### Molecular identification

Bacteria colony PCR amplification was performed using a Maxime PCR PreMix kit (iNtRON Biotechnology, Korea). The PCR mixture contained 1.0 μl of each of the 27F and 1492R primers [28], 1.0 μl diluted bacterial isolates, and 22.0 μl distilled water. Thermal cycling consisted of 95°C for 10 min, and 35 cycles of 95°C for 40 sec, 55°C for 40 sec, 72°C for 60 sec, and then incubation at 72°C for 5 min. The PCR products were electrophoresed through a 1% agarose gel and purified using Expin^TM^ PCR Purification Kits (GeneAll Biotechnology, Korea) following the manufacturer’s instructions. Purified PCR products were sequenced using an ABI3700 automated DNA sequencer at Macrogen (Seoul, Korea). The sequences were aligned using MAFFT [29] and edited manually with MEGA v.5.0 [30]. Phylogenetic trees were constructed using the neighbor-joining method with 1000 bootstrap replications. Reference sequences were retrieved from the EzBioCloud database [31]. Based on species identification, the number of species were compared between each part using Kruskal-Wallis rank sum test and Dunn’s test as a post hoc test adjusted by the false discovery rate of Benjamini and Hochberg [32]. Sequences generated from the study were deposited in Genbank under accession numbers KY992547-KY992562.

### Co-culture of bacteria with isolates of PM and molds

To prepare bacterial inocula for the co-culture experiment, bacterial strains were recovered from the stocks we isolated and stored on TSA medium at 30°C. Co-culture was performed using 60 mm Petri dishes containing ‘*Tricholoma matsutake*’ Media (TMM) (glucose 20 g/L, yeast extract 1.5 g/L, soytone 1.5 g/L, and agar 20 g/L) which is one of the best media for the growth of PM [33, 34]. We used a single PM strain that was obtained from a PM fruiting body (KMRB 12100405), which was deposited at the Korea Mushroom Resource Bank (Seoul, South Korea). To inoculate PM strains, the strain was cultured in potato dextrose broth (Difco, USA), then filtered and washed with sterilized distilled water. The washed PM tissues were then ground in 30 ml of sterilized distilled water using a homogenizer, and 20 μl of PM suspension was inoculated at the center of each 60 mm Petri dish. Using a sterile inoculating loop (SPL Life Science, Korea), representative strains selected from each bacterial species were streaked along a 30 mm line situated 15 mm away from the center of the Petri dish (Fig 1B). Plates were sealed and incubated at 25°C for one month. The diameter of PM mycelia on each plate was then measured at two different locations.

Bacterial strains were co-cultured with molds to assess the antifungal activity of bacterial species. For covering various molds that present naturally in PM fruiting bodies, we used 17 isolates of mold species that were originated from PM fruiting bodies and deposited at the Seoul National University Fungal Collection (SFC) (Table 1). Similar to the bacteria-PM strain co-culture experiment, an agar plug of each mold was placed in the center of a 90 mm Petri dish containing potato dextrose agar (PDA; Difco, USA). Bacterial strains were streaked along a 40 mm line situated 25 mm away from the center of each 90 mm Petri dish. All of the co-culture experiments were conducted in triplicate for each condition. Significance of difference was tested using pairwise t-tests with multiple test corrections by the false discovery rate of Benjamini and Hochberg [32].

**Table 1:** Enzyme activity of bacteria and molds isolated from the fruiting body of PM.

Kingdom	Phylum	Species	Chi	eGl	bGl	Prt	Lip
Bacteria	Actinobacteria	*Brevibacterium epidermidis*	-	-	-	-	-
		*Br*. *iodinum*	-	-	-	-	-
		*Dietzia aurantiaca*	-	+	+	-	+
		*Mycetocola lacteus*	-	-	-	-	-
		*Rhodococcus degradans*	-	-	-	-	-
	Firmicutes	*Bacillus toyonensis*	-	+	+	+	+
		*Paenibacillus taichungensis*	+	+	+	+	-
		*Staphylococcus hominis*	-	-	-	+	-
		*Staphylococcus lentus*	-	-	-	-	-
	Proteobacteria	*Cedecea neteri*	-	-	-	-	-
		*Comamonas koreensis*	-	-	-	+	+
		*Ewingella americana*	-	-	-	-	+
		*Pseudomonas endophytica*	-	+	+	-	-
		*Ps*. *koreensis*	-	+	+	-	+
		*Serratia marcescens*	+	+	+	+	+
		*Stenotrophomonas maltophilia*	+	+	+	+	+
Fungi	Ascomycota	*Penicillium bissettii*	-	+	+	+	-
		*P*. *crustosum*	-	+	+	+	+
		*P*. *daleae*	-	+	+	+	-
		*P*. *oxalicum*	-	+	+	+	-
		*P*. *polonicum*	-	-	-	+	-
		*Penicillium* sp.1	-	-	-	-	-
		*Penicillium* sp.2	-	-	-	-	-
		*Sarocladium kiliense*	-	+	+	+	+
		*Trichoderma songyi*	-	-	-	-	+
		*Trichoderma* sp.	-	-	-	-	-
	Zygomycota	*Absidia* sp.	-	-	-	+	-
		*Cunninghamella elegans*	-	-	-	-	-
		*Mucor irregularis*	-	-	-	-	-
		*Mu*. *silvaticus*	-	-	-	-	-
		*Mucor* sp.	-	-	-	-	-
		*Simplicillium lamellicola*	-	-	-	-	-
		*Umbelopsis nana*	-	+	+	-	+

Chi: chitinase, eGl: endoglucanase, bGl: β-glucosidase, Prt: protease, Lip: lipase

### Enzyme activity assays

We assessed the activity of five different enzymes associated with fungal cell wall component—chitinase, cellulase (endoglucanase and β-glucosidase), protease, and lipase—for bacteria and molds. Chitinase activity was assessed based on the Roberts and Selitrennikoff method [35]. Colloidal chitin was prepared using shrimp shell chitin (Sigma-Aldrich, USA) following the methods of Roberts and Selitrennikoff [35]. Endoglucanase and β-glucosidase activities were assayed using Mandels’ medium with 1% carboxymethylcellulose (Sigma-Aldrich, USA) and 0.5% D-cellobiose (Sigma-Aldrich, USA), respectively [36]. Extracellular protease activity was assayed using yeast extract agar (Difco, USA) with 1.5% skim milk (Difco, USA) [37]. Lipase activity was assayed using 9% agar with Bacto peptone (10 g/L), NaCl (5 g/L), CaCl_2_·H_2_O (0.1 g/L), and 1% Tween80 [38]. The association between presence of enzyme activity and effect on fungal growth was tested using Fisher’s exact test.

## Results

### Microbial diversity associated with the fruiting body of PM

**Fig 2:**
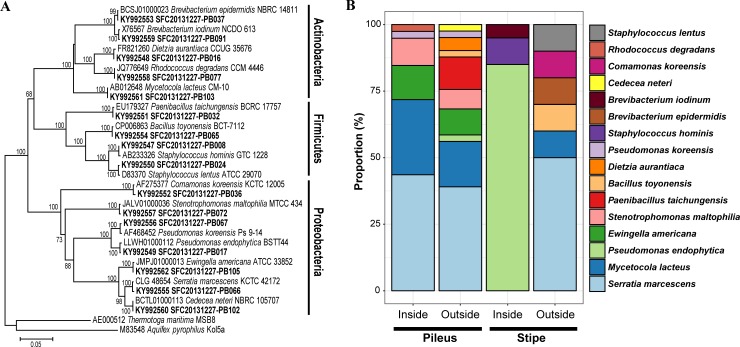
Bacterial diversity isolated from the fruiting body of PM. (A) Phylogenetic analysis based on 16S rRNA sequences using neighbor-joining method with 1000 bootstrap replicates. (B) Relative abundance of bacterial species in different parts of the fruiting body.

A total of 110 bacterial strains were isolated from 240 fruiting body sections (48 pieces × 5 fruiting bodies). 16S rDNA sequence analysis for 110 bacterial isolates revealed a total of 16 different species of bacteria (Fig 2A). All 16S rDNA sequences had high similarity with the type species (99.6–100%) and final identification was assigned based on the phylogenetic tree. The highest number of species were found in the Proteobacteria (7 species), followed by the Actinobacteria (5 species) and the Firmicutes (4 species). In a total of bacterial isolates, *Serratia marcescens* was the most dominant species of bacteria (34.5%), followed by *Mycetocola lacteus* (17.3%) and *Pseudomonas endophytica* (16.4%). Bacterial diversity varied largely among parts of the fruiting body: the outside of the pileus harbored the largest number (10 species), while the inside of the stipe harbored the least (3 species) (Fig 2B). The number of bacterial species was significantly higher in the pileus compared to the stipe (inside pileus > inside stipe: *P* = 0.03; outside pileus > inside stipe: *P* = 0.01; inside pileus > outside stipe: *P* = 0.04; outside pileus > outside stipe: *P* = 0.02), while the number of bacterial species was comparable between pileus parts (*P* = 0.64) and between stipe parts (*P* = 0.98) (S1 Table). In a total of bacterial isolates from each fruiting body parts, *Se*. *marcescens* was dominant at all parts on and within the fruiting body (39.0–50.0%), except inside the stipe, where *Ps*. *endophytica* was dominant (85.0%) (Fig 2B).

### Bacterial effect on the growth of PM and molds

**Fig 3:**
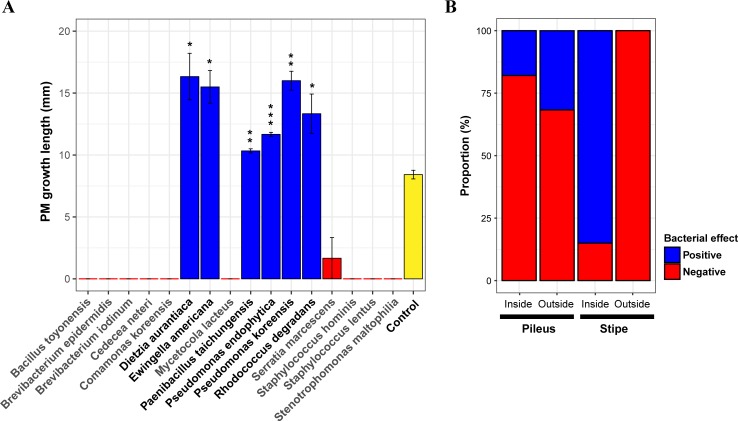
Effect of bacteria on the growth of PM. (A) Mycelial growth of PM co-cultured with bacterial species. Pairwise t-test (one-tailed) was conducted to compare between growth promotion in co-culture and control. Significance of test was corrected by the false discovery rate of Benjamini and Hochberg. (B) Composition of bacterial effect in each part of the fruiting body.

Among the 16 bacterial species isolated from PM fruiting bodies, ten appeared to have a strong negative effect on the mycelial growth of PM, as little or no growth (0–3 mm) was observed when these species were present (Fig 3A). The bacterial species that appeared inhibitory to PM growth included some of the species found to dominate the fruiting bodies such as *Se*. *marcescens*, *My*. *lacteus*, and *Stenotrophomonas maltophilia*. In contrast, six bacterial species stimulated PM mycelial growth significantly (growth change: 123–194%) (Fig 3A; S2 Table). This group of growth-promoting bacteria included *Dietzia aurantiaca*, *Ewingella americana*, *Paenibacillus taichungensis*, *Pseudomonas* spp., and *Rhodococcus degradans*. *D*. *aurantiaca* (growth change: 194%) and *Ps*. *koreensis* (growth change: 190%) had the largest growth-promoting effect (S2 Table). Based on the results of the co-culture experiment, the distribution of bacterial effects was assigned to different locations of the fruiting body: bacteria that had an inhibitory effect on PM were dominant in most areas of the fruiting body (68.3–100%) while growth-promoting bacteria were dominant inside the stipe (85.0%) (Fig 3B).

**Fig 4:**
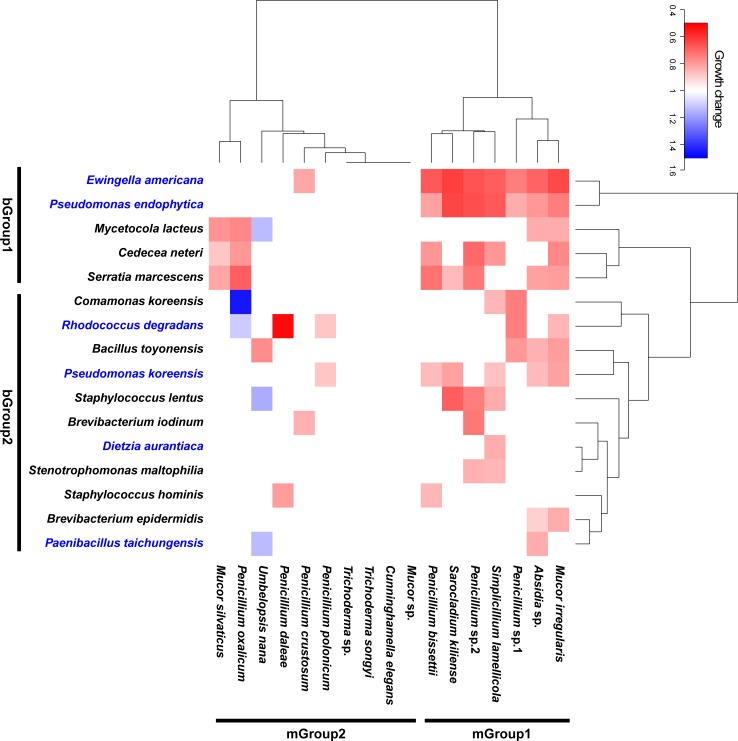
Heatmap plot for growth change of molds co-cultured with bacteria isolated from the fruiting body of PM. Growth change is represented as color with significance detected by a pairwise t-test. The bacterial names in blue color represent growth-promoting bacteria. bGroup1: bacterial group showing a wide range of antifungal activity, bGroup2: bacterial group with a narrow range of influence, mGroup1: generally suppressed mold group, mGroup2: specifically influenced mold group.

Among the 272 bacteria-mold pairings (16 bacteria × 17 molds) we cultured, a change in growth was significant for 66 pairs (Fig 4; S2 Table): 61 pairs exhibited suppressed growth of mycelial growth of mold species, while five pairs showed growth promotion. No single bacterial species affected all molds (Fig 4). Five of the 16 bacterial species showed a wide range of antifungal activity (bGroup 1) while the remaining 11 species had a narrower range of influence (bGroup 2). *Ewingella americana* had a suppressive effect on the widest range of mold species (8 molds; growth change: 62–82%), followed by *Se*. *marcescens* (7 molds; growth change: 68–87%) and *Ps*. *endophytica* (7 molds; growth change: 63–84%). Growth response of our 17 test molds to the 16 bacteria separated into two groups: a generally suppressed group of molds (mGroup 1) and a specifically influenced group of molds (mGroup 2) (Fig 4). mGroup 1 was suppressed by five to nine bacterial species, and included *Absidia* sp., *Mucor irregularis*, *Penicillium bissettii*, *Penicillium* sp.1, *Penicillium* sp.2, *Sarocladium kiliense*, and *Simplicillium lamellicola*. Mold growth belonging to mGroup 2 was influenced by two to five species, while some bacteria showed growth promotion of molds. Growth of *Pe*. *oxalicum* was significantly increased by *Comamonas koreensis* (145%) and *R*. *degradans* (113%), and growth of *Umbelopsis nana* was significantly increased by *My*. *lacteus* (Growth change: 113%), *Pa*. *taichungensis* (Growth change: 113%), and *Staphylococcus lentus* (Growth change: 116%) (S2 Table).

### Enzyme activity assays

Among the 16 bacterial species we assayed for five different enzymes (chitinase, endoglucanase, β-glucosidase, protease, and lipase), ten species showed enzyme activity. Only two species, *Se*. *marcescens* and *Stenotrophomonas maltophilia*, exhibited activity for all enzymes tested. Chitinase activity was found in three species. Seven species showed cellulase activity. Protease activity was detected from six species and lipase activity from seven species (Table 1).

Nine of 17 mold species we assayed exhibited enzyme activity, and *Sa*. *kiliense* and *Pe*. *crustosum* exhibited enzyme activity for all enzymes tested except chitinase. In the Ascomycota, four *Penicillium* species showed cellulase and protease activities. Among seven species in the Zygomycota, only two species showed enzyme activity: *Absidia* sp. had protease activity and *U*. *nana* had cellulase and lipase activities (Table 1).

A Fisher’s exact test showed that there was no relationship between any of the enzyme activities and effect on mycelial growth of PM. Moreover, neither presence of enzyme activity or sharing the same enzyme activity between bacteria and molds significantly influenced growth of mold mycelia.

## Discussion

Diverse bacteria have been reported from PM fruiting bodies by culture dependent and high-throughput sequencing methods. Recently, *Alcaligenes* sp., *Ew*. *americana*, *Pseudomonas* sp., *Serratia* sp., *Sphingobacerium* sp., and *Stenotrophomonas* sp. have been isolated from the fruiting bodies of PM in China [27]. Pyrosequencing showed that *Pseudomonas* and *Stenotrophomonas* were abundant in the fruiting bodies of PM [26]. Our results indicate that *Se*. *marcescens* was the most abundant species found in PM fruiting bodies, followed by *My*. *lacteus*, *Ps*. *endophytica*, *Ew*. *americana*, and *Stenotrophomonas maltophilia* (Fig 2B). Although our sampling site in Korea is 2,600 km from the Li et al. [26] site in China, similar studies support the idea that the bacterial community is affected by the mushroom identity where bacteria live [4]. The PM fruiting body harbored different dominant bacterial genera compared to the bacterial genera found in the soil of the fairy ring (*e*.*g*. *Burkholderia*, *Mycobacterium*, and *Paenibacillus*) [25]. Although part of these community differences probably resulted from the cultural state of bacterial species used in the comparison, our results indicate that the abundance of several bacterial taxa differ considerable between fruiting body and surrounding soil [4, 39]. Interestingly, the bacterial community inside the stipe was distinct from other areas of the fruiting body (Fig 1B). Most bacteria isolated from inside the stipe were *Ps*. *endophytica*, which was first reported as an endophytic bacterium in the stem and root of potato [40]. Several bacterial genera detected in this study have been found in a wide variety of fruiting bodies: *Agaribus bisporus* [41], *Armillaria mellea* [42], *Cantharellus* spp. [43], *Tuber borchii* [44], and *Suillus grevillea* [45]. This suggests that some fruiting body bacteria may adapt to the fruiting body environment.

Many studies have shown that bacteria associated with mushrooms can positively influence the physiology of fungi [7, 46, 47]. For example, *Ps*. *putida* induces initiation of fruiting body formation in *Agaricus bisporus* [8]. In addition, *Streptomyces* sp. and *Ps*. *fluorescence* promote hyphal growth and mycorrhizal formation in *Amanita muscaria* and *Laccaria bicolor*, respectively [48–50]. In *Tuber melanosporum*, the bacterial community is involved in fruiting body maturation [5] and aroma formation [51]. Similarly, six bacteria had a significant positive effect on the mycelial growth of PM (Fig 3A). Among these, species in *Paenbacillus*, *Pseudomonas*, and *Rhodococcus* are well-known mycorrhiza helper bacteria (MHB) [7]. Although the distribution of growth-promoting bacteria was not restricted to the inside of the PM fruiting bodies we sampled, the proportion of growth-promoting bacteria was higher inside the stipe (85.0%) compared to other areas, primarily due to the high abundance of *Ps*. *endophytica* (Fig 3B). In contrast, except to six bacteria, most bacteria associated with the PM fruiting body had a strong negative effect on the mycelial growth of PM (Fig 3A). These results are consistent with those of Danell et al. [52] and Varese et al. [45] who found that bacteria isolated from the fruiting bodies of *Cantharellus cibarius* and *Suillus grevillei* suppressed hyphal growth of the fungi. These phenomena imply that most bacteria that have a negative impact on PM growth may use the PM fruiting body as a nutrient source.

Indirect positive effects of bacteria on the PM were tested by measuring the influence of bacteria on the growth of molds (Fig 4; S2 Table). Molds are frequently detected from fungal fruiting bodies [16, 18, 53] and some molds are known as pathogens [54–56] or early decomposers of dead fruiting bodies [57]. Because of the diversity of enzyme activities among the molds (Table 1), it is likely that the presence of molds often introduces some negative effects on the maintenance of the PM fruiting body. Some bacteria associated with the PM fruiting body do appear to act as a biocontrol agent against some mold species [58–60]. For example, *Pseudomonas* suppresses the growth of molds in the *Tuber borchii* [44]. Interestingly, in our study, the co-culture results for the bacteria-mold pairings we tested showed that antifungal activity was different among bacterial species (Fig 4). *Ewingella americana*, *Ps*. *endophytica*, and *Se*. *marcescens* were the strongest suppressors of a wide range of molds (growth change: 62.6–87.0%). Molds could be separated into two groups according to suppression pattern: a generally suppressed group (mGroup 1) and a specifically influenced group (mGroup 2) (Fig 4). The generally suppressed group was composed of *Absidia*, *Mucor*, *Penicillium*, and *Sarocladium*, which were abundant in environments associated with PM, including the fruiting body [16], ectomycorrhiza [61], and soil [62]. Thus, it is possible that frequently detected molds may be more likely to be suppressed by abundant fruiting body bacteria (Fig 4). In the case of the specifically influenced group, selective interactions may exist. Interestingly, some bacteria promoted growth in *Pe*. *oxalicum* and *U*. *nana*, although the mechanisms of this growth promotion are unclear. However, *Umbelopsis* is frequently found in the fairy rings of PM [25, 63], and its metabolites promote growth of PM [64]. Thus, there appears to be a strong relationship between *Umbelopsis* and PM, and this relationship appears to be influenced by the growth promotion effect of bacteria on *Umbelopsis*. The interaction is likely complex, thus the extent of mold influence on PM requires a thorough understanding of the microbial interactions in the fruiting body, and remains to be elucidated.

Many of the bacteria isolated from PM fruiting bodies showed high enzyme activity, which suggests mycophagy in these bacteria is associated with PM fruiting bodies, a phenomenon that has been documented in other studies [43, 65–67]. Our enzyme activity assays suggested that fruiting body bacteria are able to degrade fungal cell walls (Table 1). For example, *Pa*. *taichungensis*, *Se*. *marcescens*, and *Stenotrophomonas maltophilia* showed chitinase and cellulase activities as reported previously [13, 68, 69]. Given that carbohydrates and proteins are the major components of the PM [70], high enzyme activity for chitinase, cellulase, and protease indicate that some bacteria may use the PM fruiting body as a nutrient source. On the other hand, bacterial enzymes (*e*.*g*. chitinase and protease) are often responsible for the antifungal activity of bacteria [71–73]. In our study, however, enzyme activity was not associated with growth of PM. Thus, any bacterial influence on the growth of PM may be due to antifungal agents other than those assessed in this study.

The common enzyme activities observed in bacteria and molds suggest that bacteria and molds compete for the same nutrient sources. Therefore, antifungal activity may be high in the bacteria-mold pairs that shared same enzyme activity. However, our results did not show any relationship between enzyme activity and antifungal activity, which suggests that bacteria may provide indirect positive effects to PM as a biocontrol agent, irrespective of nutrient competition. Bacteria often use nutrients provided from associated fungi (*e*.*g*. sugar and amino acid), rather than consuming the fungal cell wall [74, 75]. If PM provides nutrients for bacteria, then maintenance of PM would be advantageous to these bacteria, which may explain why bacteria suppress molds.

Most bacteria in environmental samples are unculturable; less than 1% of bacteria grow in artificial media [76]. Since our study focused on the complex interactions between PM and bacteria, and required living bacterial strains rather than simply presence/absence of bacterial species indicated by DNA analysis, our data are limited to this small fraction of culturable bacteria. It is likely that we have only scratched the surface in uncovering the complex interactions between bacteria and PM. Evidence for this is highlighted in one metagenomic study that found 40 different prokaryotic phyla in PM fruiting bodies (only 16 bacterial species were cultured in this study) [26]. Future studies that include uncultured bacterial strains identified from metagenomic studies are needed; however, this will require extensive testing of culture methods for strains that do not grow in standard conditions or standard media, and are beyond the scope the present study.

In conclusion, while many bacterial species have a negative effect on the growth of PM mycelia and likely exploit the fruiting body of PM as a nutrient source, others have a positive influence on PM growth. We found direct positive effects of bacteria (*e*.*g*. *Ewingella* and *Pseudomonas*) on the mycelial growth of PM, as well as indirect positive effects of bacteria as biocontrol agents against molds. The mechanisms associated with these bacterial effects, i.e. specific metabolites and optimal growth conditions, remain to be elucidated and a better understanding of these factors may lead to successful cultivation of PM.

## Supporting information

S1 Table The number of bacterial isolate from each part of PM fruiting bodies. (DOCX)

S2 Table Average growth change of PM and molds co-cultured with bacteria. Significance of difference was tested using pairwise t-test adjusted by the false discovery rate of Benjamini and Hochberg. Significantly different growth changes are highlighted in grey. Significant growth promotions are indicated by bolded numbers.
(DOCX)
