# Supplementary material for: Effect of fruiting body bacteria on the growth of Tricholoma matsutake and its related molds
Source: PLoS One. 2018 Feb 8;13(2):e0190948. doi: 10.1371/journal.pone.0190948 (PMC5805168; doi:10.1371/journal.pone.0190948)
Supplement: S1 Table — (DOCX) [file pone.0190948.s001.docx]

**S1 Table. The number of bacterial isolate from each part of PM fruiting bodies.**

| **Bacteria name** | **Pileus inside** | | | | |  | **Pileus outside** | | | | |  | **Stipe inside** | | | | |  | **Stipe outside** | | | | |
| --- | --- | --- | --- | --- | --- | --- | --- | --- | --- | --- | --- | --- | --- | --- | --- | --- | --- | --- | --- | --- | --- | --- | --- |
|  | ***s1** | **s2** | **s3** | **s4** | **s5** |  | **s1** | **s2** | **s3** | **s4** | **s5** |  | **s1** | **s2** | **s3** | **s4** | **s5** |  | **s1** | **s2** | **s3** | **s4** | **s5** |
| *Bacillus toyonensis* | 0 | 0 | 0 | 0 | 0 |  | 0 | 0 | 1 | 0 | 0 |  | 0 | 0 | 0 | 0 | 0 |  | 0 | 0 | 1 | 0 | 0 |
| *Brevibacterium epidermidis* | 0 | 0 | 0 | 0 | 0 |  | 0 | 0 | 0 | 0 | 0 |  | 0 | 0 | 0 | 0 | 0 |  | 0 | 1 | 0 | 0 | 0 |
| *Brevibacterium iodinum* | 0 | 0 | 0 | 0 | 0 |  | 0 | 0 | 0 | 0 | 0 |  | 0 | 0 | 0 | 1 | 0 |  | 0 | 0 | 0 | 0 | 0 |
| *Cedecea neteri* | 0 | 0 | 0 | 0 | 0 |  | 0 | 1 | 0 | 0 | 0 |  | 0 | 0 | 0 | 0 | 0 |  | 0 | 0 | 0 | 0 | 0 |
| *Comamonas koreensis* | 0 | 0 | 0 | 0 | 0 |  | 0 | 0 | 0 | 0 | 0 |  | 0 | 0 | 0 | 0 | 0 |  | 0 | 0 | 0 | 1 | 0 |
| *Dietzia aurantiaca* | 0 | 0 | 0 | 0 | 0 |  | 0 | 0 | 1 | 1 | 0 |  | 0 | 0 | 0 | 0 | 0 |  | 0 | 0 | 0 | 0 | 0 |
| *Ewingella americana* | 1 | 1 | 1 | 2 | 0 |  | 2 | 1 | 0 | 0 | 1 |  | 0 | 0 | 0 | 0 | 0 |  | 0 | 0 | 0 | 0 | 0 |
| *Mycetocola lacteus* | 3 | 2 | 1 | 2 | 3 |  | 0 | 0 | 2 | 3 | 2 |  | 0 | 0 | 0 | 0 | 0 |  | 1 | 0 | 0 | 0 | 0 |
| *Paenibacillus taichungensis* | 0 | 0 | 0 | 0 | 0 |  | 1 | 1 | 1 | 1 | 1 |  | 0 | 0 | 0 | 0 | 0 |  | 0 | 0 | 0 | 0 | 0 |
| *Pseudomonas endophytica* | 0 | 0 | 0 | 0 | 0 |  | 0 | 1 | 0 | 0 | 0 |  | 4 | 6 | 2 | 2 | 3 |  | 0 | 0 | 0 | 0 | 0 |
| *Pseudomonas koreensis* | 0 | 0 | 0 | 0 | 1 |  | 0 | 0 | 1 | 0 | 0 |  | 0 | 0 | 0 | 0 | 0 |  | 0 | 0 | 0 | 0 | 0 |
| *Rhodococcus degradans* | 0 | 1 | 0 | 0 | 0 |  | 0 | 0 | 0 | 0 | 0 |  | 0 | 0 | 0 | 0 | 0 |  | 0 | 0 | 0 | 0 | 0 |
| *Serratia marcescens* | 3 | 3 | 6 | 1 | 4 |  | 5 | 3 | 2 | 2 | 4 |  | 0 | 0 | 0 | 0 | 0 |  | 1 | 2 | 0 | 0 | 2 |
| *Staphylococcus hominis* | 0 | 0 | 0 | 0 | 0 |  | 0 | 0 | 0 | 0 | 0 |  | 0 | 0 | 1 | 0 | 1 |  | 0 | 0 | 0 | 0 | 0 |
| *Staphylococcus lentus* | 0 | 0 | 0 | 0 | 0 |  | 0 | 0 | 0 | 0 | 0 |  | 0 | 0 | 0 | 0 | 0 |  | 1 | 0 | 0 | 0 | 0 |
| *Stenotrophomonas maltophilia* | 0 | 1 | 1 | 0 | 2 |  | 0 | 0 | 0 | 3 | 0 |  | 0 | 0 | 0 | 0 | 0 |  | 0 | 0 | 0 | 0 | 0 |

*s: sample number of fruiting bodies of *T. matsutake*
