# Supplementary material for: Effect of fruiting body bacteria on the growth of Tricholoma matsutake and its related molds
Source: PLoS One. 2018 Feb 8;13(2):e0190948. doi: 10.1371/journal.pone.0190948 (PMC5805168; doi:10.1371/journal.pone.0190948)
Supplement: S2 Table — Significance of difference was tested using pairwise t-test adjusted by the false discovery rate of Benjamini and Hochberg. Significantly different growth changes are highlighted in grey. Significant growth promotions are indicated by bolded numbers. (DOCX) [file pone.0190948.s002.docx]

**S2 Table. Average growth change of PM and molds co-cultured with bacteria.** Significance of difference was tested using pairwise t-test adjusted by the false discovery rate of Benjamini and Hochberg. Significantly different growth changes are highlighted in grey. Significant growth promotions are indicated by bolded numbers.

|  | ***B. toyonensis*** | ***B. epidermidis*** | ***B. iodinum*** | ***C. neteri*** | ***C. koreensis*** | ***D. aurantiaca*** | ***E. americana*** | ***M. lacteus*** | ***P. taichungensis*** | ***P. endophytica*** | ***P. koreensis*** | ***R. degradans*** | ***S. marcescens*** | ***S. hominis*** | ***S. lentus*** | ***S. maltophilia*** |
| --- | --- | --- | --- | --- | --- | --- | --- | --- | --- | --- | --- | --- | --- | --- | --- | --- |
| **PM** | 0.00 | 0.00 | 0.00 | 0.00 | 0.00 | **1.94** | **1.84** | 0.00 | **1.23** | **1.39** | **1.90** | **1.58** | 0.20 | 0.00 | 0.00 | 0.00 |
| ***Absidia* sp.** | 0.85 | 0.91 | 0.90 | 0.83 | 0.90 | 0.92 | 0.69 | 0.83 | 0.83 | 0.80 | 0.87 | 1.10 | 0.82 | 1.06 | 0.91 | 0.94 |
| ***C. elegans*** | 1.03 | 1.10 | 1.06 | 1.04 | 1.16 | 1.05 | 0.86 | 0.93 | 0.91 | 0.99 | 1.03 | 0.99 | 0.96 | 1.04 | 1.01 | 1.05 |
| ***M. irregularis*** | 0.81 | 0.83 | 0.83 | 0.77 | 0.87 | 0.94 | 0.64 | 0.84 | 0.94 | 0.74 | 0.82 | 0.86 | 0.81 | 1.04 | 0.91 | 0.97 |
| ***M. silvaticus*** | 0.95 | 1.00 | 0.92 | 0.89 | 0.97 | 0.85 | 0.69 | 0.78 | 0.81 | 0.87 | 0.97 | 0.95 | 0.82 | 0.96 | 0.81 | 0.84 |
| ***Mucor* sp.** | 0.90 | 1.00 | 0.98 | 0.98 | 1.00 | 0.96 | 0.82 | 0.89 | 0.93 | 0.96 | 0.98 | 1.04 | 0.96 | 1.03 | 0.92 | 0.98 |
| ***P. bissettii*** | 0.91 | 0.95 | 0.85 | 0.80 | 0.94 | 0.96 | 0.68 | 0.87 | 0.86 | 0.82 | 0.87 | 0.98 | 0.72 | 0.86 | 0.91 | 0.84 |
| ***P. crustosum*** | 0.90 | 0.88 | 0.85 | 0.87 | 0.87 | 1.02 | 0.83 | 0.90 | 1.00 | 0.92 | 0.87 | 0.95 | 0.87 | 0.93 | 1.02 | 0.96 |
| ***P. daleae*** | 0.91 | 0.82 | 0.85 | 0.79 | 0.90 | 1.06 | 0.89 | 0.90 | 0.92 | 1.00 | 0.95 | 0.53 | 0.85 | 0.81 | 1.03 | 0.81 |
| ***P. oxalicum*** | 0.95 | 1.21 | 1.01 | 0.79 | **1.45** | 1.22 | 0.82 | 0.76 | 0.86 | 0.96 | 0.85 | **1.11** | 0.69 | 0.93 | 1.10 | 0.85 |
| ***P. polonicum*** | 0.94 | 0.95 | 0.98 | 0.92 | 0.96 | 0.97 | 0.91 | 0.96 | 1.01 | 0.89 | 0.88 | 0.88 | 0.93 | 0.92 | 0.96 | 0.95 |
| ***Penicillium* sp.1** | 0.79 | 0.81 | 0.81 | 0.86 | 0.74 | 0.99 | 0.74 | 1.02 | 1.06 | 0.84 | 0.74 | 0.75 | 0.88 | 0.99 | 0.99 | 0.91 |
| ***Penicillium* sp.2** | 0.72 | 0.85 | 0.74 | 0.70 | 0.77 | 0.80 | 0.67 | 0.97 | 0.98 | 0.66 | 0.74 | 0.96 | 0.74 | 1.04 | 0.75 | 0.85 |
| ***S. kiliense*** | 0.71 | 0.96 | 0.94 | 0.85 | 0.89 | 0.75 | 0.63 | 0.94 | 0.93 | 0.63 | 0.81 | 0.95 | 0.87 | 0.87 | 0.68 | 0.93 |
| ***S. lamellicola*** | 0.97 | 0.97 | 0.91 | 0.79 | 0.85 | 0.83 | 0.68 | 0.89 | 0.88 | 0.67 | 0.87 | 0.95 | 0.77 | 1.05 | 0.83 | 0.85 |
| ***T. songyi*** | 0.83 | 0.91 | 0.99 | 0.90 | 0.96 | 0.98 | 0.95 | 0.87 | 0.90 | 0.98 | 0.98 | 0.92 | 0.90 | 0.92 | 0.97 | 0.89 |
| ***Trichoderma* sp.** | 0.91 | 0.94 | 0.93 | 0.93 | 0.91 | 1.00 | 0.99 | 0.92 | 0.87 | 0.99 | 0.91 | 1.03 | 0.95 | 1.02 | 0.98 | 0.87 |
| ***U. dimorpha*** | 0.77 | 0.95 | 1.02 | 0.90 | 0.98 | 1.05 | 0.93 | **1.14** | **1.14** | 1.10 | 1.14 | 1.03 | 1.01 | 1.15 | **1.16** | 0.95 |
